# Chlamydia pneumoniae-associated pleuropericarditis: a case report and systematic review of the literature

**DOI:** 10.1186/s12890-021-01743-9

**Published:** 2021-11-22

**Authors:** Konstantinos G. Kyriakoulis, Anastasios Kollias, George E. Diakos, Ioannis P. Trontzas, Eleni Fyta, Nikolaos K. Syrigos, Garyphallia Poulakou

**Affiliations:** Third Department of Medicine, School of Medicine, National and Kapodistrian University of Athens, Sotiria Hospital, 152 Mesogion Avenue, 11527 Athens, Greece

**Keywords:** Pericarditis, Pleuritis, Pleuropericarditis, Serositis, Chlamydia pneumonia, Community acquired pneumonia, Antigenic mimicry

## Abstract

**Background:**

*Chlamydia pneumoniae* is a common cause of atypical community acquired pneumonia (CAP). The diagnostic approach of chlamydial infections remains a challenge. Diagnosis of delayed chlamydial-associated complications, involving complex autoimmune pathophysiological mechanisms, is still more challenging. *C. pneumoniae*-related cardiac complications have been rarely reported, including cases of endocarditis, myocarditis and pericarditis.

**Case presentation:**

A 40-year old female was hospitalized for pleuropericarditis following lower respiratory tract infection. The patient had been hospitalized for CAP (fever, dyspnea, chest X-ray positive for consolidation on the left upper lobe) 5 weeks ago and had received ceftriaxone and moxifloxacin. Four weeks after her discharge, the patient presented with fever, shortness of breath and pleuritic chest pain and was readmitted because of pericardial and bilateral pleural effusions (mainly left). The patient did not improve on antibiotics and sequential introduction of colchicine and methylprednisolone was performed. The patient presented impressive clinical and laboratory response. Several laboratory and clinical assessments failed to demonstrate any etiological factor for serositis. Chlamydial IgM and IgG antibodies were positive and serial measurements showed increasing kinetics for IgG. Gold standard polymerase chain reaction of respiratory tract samples was not feasible but possibly would not have provided any additional information since CAP occurred 5 weeks ago. The patient was discharged under colchicine and tapered methylprednisolone course. During regular clinic visits, she remained in good clinical condition without pericardial and pleural effusions relapse.

**Conclusions:**

*C. pneumoniae* should be considered as possible pathogen in case of pleuritis and/or pericarditis during or after a lower respiratory tract infection. In a systematic review of the literature only five cases of *C. pneumoniae* associated pericarditis were identified. Exact mechanisms of cardiovascular damage have not yet been defined, yet autoimmune pathways might be implicated.

**Supplementary Information:**

The online version contains supplementary material available at 10.1186/s12890-021-01743-9.

## Background

*Chlamydia pneumoniae* is a common cause of atypical lower respiratory tract infection [1]. Cardiac complications related to *C. pneumoniae* infection have been rarely reported. These reports include cases of endocarditis, myocarditis or pericarditis during or after *C. pneumoniae* pneumonia [2–11]. Atherosclerosis has also been related to *C. pneumoniae* infection [12–14]. We present the case of a 40-year old woman who suffered serositis (pleuropericarditis) after being treated for community acquired pneumonia (CAP). The diagnosis of *C. pneumoniae* serositis was established based on the kinetics of chlamydial antibodies over serial measurements. A systematic review of the literature was also performed in line with the PRISMA recommendations (www.prisma-statement.org) to detect similar cases.

## Case presentation

A 40-year old female was transferred to our Department of Medicine for the assessment and treatment of pleuropericarditis following lower respiratory tract infection. Her body mass index was 25.7 kg/m^2^ and her personal history was positive for current smoking (~ 10 cigarettes/day, 40 pack years) and arterial hypertension (occasional use of amlodipine 5 mg). The present history of the patient begins 5 weeks ago with a hospitalization in a Respiratory Department for CAP (fever, dyspnea, chest X-ray positive for consolidation on the left upper lobe; Additional File 1). The patient received ceftriaxone and moxifloxacin for 10 days without any medical report on causative pathogen. Four weeks after her discharge the patient presented with fever, shortness of breath especially in exertion and pleuritic chest pain and was admitted to a Respiratory Department where bilateral pleural (mainly left) and mild pericardial effusion were found. Pericardial friction rub was not identified. Her blood tests revealed increased neutrophils and indices of inflammation (Table 1, Additional File 1). Piperacillin/tazobactam and linezolid were initially administered. Thoracocentesis on the left side revealed neutrophilic exudate with pH 7.40. Despite antibiotic treatment, patient was not improved. Moreover C-reactive protein (CRP), pleural and pericardial effusions remained unchanged. Procalcitonin, high-sensitive troponin and brain natriuretic peptide levels were within normal limits during hospitalization. Blood and sputum cultures were negative. Extensive rheumatological workup was negative. In electrocardiography sinus tachycardia (112 bpm), frequent premature ventricular contractions, periods of ventricular trigeminy and inverted T-wave in V1–V6 were observed. Echocardiography was positive for mild mitral and triscupid valve regurgitation and mild pericardial effusion, without any sign of hemodynamic decompensation. Computed tomography (CT) imaging (including CT pulmonary angiogram) did not reveal pulmonary embolism or pathological findings from lung parenchyma and was positive only for the presence of pleural and pericardial effusions (Additional File 1). Pulmonologists administered ibuprofen 600 mg three times daily with the indication of idiopathic pleuropericarditis. The patient presented allergic reaction to ibuprofen and was transferred to our Department of Medicine on day (D) 12. Upon her admission, the patient presented with diffuse maculo-papular rash that was attributed to the allergic reaction to ibuprofen. Ibuprofen was withdrawn and IV methylprednisolone 40 mg once daily was initiated. Antibiotic treatment was suspended due to the lack of evidence of bacterial infection. Due to sinus tachycardia and premature ventricular complexes metoprolol 25 mg twice daily was administered. Patient’s file was reviewed and pericarditis investigation and differential diagnosis was conducted according to current guidelines [15]. The patient fulfilled two out of four major criteria (pericardial chest pain and new pericardial effusion), along with one of the additional supporting findings (elevated CRP, white blood cells and erythrocyte sedimentation rate), rendering the diagnosis of pericarditis evidence-based [15]. Colchicine 0.5 mg once daily (patient’s weight 66 kg) was initiated, for the treatment of pericarditis under investigation. The patient presented clinical improvement and CRP dropped to nearly normal (Additional File 1). Methylprednisolone was stopped after 3 days, as it was administered to treat the allergic reaction to ibuprophen. Low grade fever and pleuritic chest pain recurred rapidly, along with rising CRP levels. A new chest X-ray revealed bilateral pleural effusion but this time mainly on the right side. A repeat thoracocentesis revealed neutrophilic exudate with pH 7.40 and in a new chest CT the only new finding was the reduction of the left pleural effusion in combination with the increase of the right sided pleural effusion. Empirical initiation of meropenem was decided (Additional File 1). Clinical and laboratory findings did not improve with the addition of antibiotics; thus, antibiotics were suspended due to lack of evidence in favor of a bacterial cause for the new fever and IV methylprednisolone 40 mg once daily was again initiated.

**Table 1:** Characteristics of cases with Chlamydia pneumoniae-associated pericarditis after systematic literature search

Case	1	2	3	4	5	6
Study	Kyriakoulis et al (*present study*)	Oztek Celebi et al. [7]	Suesaowalak et al. [8]	Rýzlová et al. [9]	Tenenbaum et al. [10]	Zver et al. [11]
Year	2020	2020	2008	2008	2005	1997
Country	Greece	Turkey	Thailand	Czech Republic	Germany	Slovenia
Age (years)	40	13	11	52	13	27
Sex	Female	Male	Male	Male	Female	Male
Main diagnosis	Pleuropericarditis	Pericarditis	Myopericarditis	Pericarditis	Pericarditis	Pericarditis
Signs/Symptoms	Fever Shortness of breath after excessive physical activity Chest pain	Chest pain Rhinitis for 3 weeks Cough for 3 weeks No fever	Fever Rash Headache Myalgia Neck pain Intermittent vomiting	Fever Shortness of breath after excessive physical activity Dry cough Chest pain	Fever Tachypnoea Shortness of breath exacerbated by exertion Throat pain Nausea	Fever Dry cough Pericardial friction rub Chest pain Cardiac tamponade
Pre-existing medical conditions	Arterial hypertension	No	No	Respiratory tract infection 2 weeks ago (clarithromycin)	Skeletal dysplasia of unknown cause, scoliosis, generalized gingivitis, mild aortic valve regurgitation	Acute myeloblastic leukemia, pancytopenia
Pleural effusion	Bilateral	Bilateral	Small bilateral	Left	No	NR
Pericardial effusion	Yes (mild)	Yes (large)	Yes (small)	Yes (large)	Yes	Yes
Pericardiocentesis	No	Yes, 1000 ml hemorrhagic	No	No	Yes, 500 ml hemorrhagic	Yes, 320 ml sanguinous exudate
ECG	Sinus tachycardia (112 bpm), PVCs, ventricular trigeminy, inverted T-wave in V1-V6	NR	Sinus tachycardia (129 bpm), low QRS voltage, inverted T-wave in III, aVF, and V1–V4	Sinus tachycardia (106 bpm), 1 mm elevations ST in II, III, aVF, V2–V6	NR	NR
WBC	13.25 × 10^9^/l (neutrophils 89%)	12.9 × 10^9^/l (neutrophils 80%)	11.6 × 10^9^/l (neutrophils 70%)	Normal	12.6 × 10^9^/l	NR
Troponin	< 1.9 pg/ml (r < 15.6)	0 ng/ml (r < 0.06)	0.9 ng/ml (r < 0.04)	Negative	NR	NR
BNP	95 pg/ml (r < 100)	NR	2.493 pg/ml (r < 100)	NR	NR	NR
CRP	8 mg/dl (r < 0.70)	719 nmol/L (r < 48)	16.18 mg/dl (r < 0.75)	302 mg/l (r NR)	20 mg/l (r NR)	NR
ESR	120 mm/h (r < 10)	13 mm/h (r < 10)	92 mm/h (r < 10)	NR	NR	NR
ANA	Negative	Negative	NR	1: 160	NR	NR
RF	< 10.2 (r < 15)	NR	NR	NR	NR	NR
Chest X-ray	Small amount of bilateral pleural effusion mainly left, cardiomegaly	Bilateral pleural effusions, lung infiltrations, cardiomegaly	Pulmonary venous congestion, small amount of bilateral pleural effusion, cardiomegaly	Left side infiltrate 3 × 2 cm	Central bilateral infiltration and an enlarged cardiac silhouette	Bronchopneumonia of right middle lobe
Echocardiography	Mild pleural effusion, normal systolic function, mild mitral and triscupid valve regurgitation	Large pericardial effusion	Mildly depressed left ventricular systolic function, EF 51%, small pericardial effusion	Pericardial effusion up to 18 mm, no signs of tamponade	Pericardial effusion	Pericardial effusion up to 27 mm, fibrous strands attached to pericardium
Chest CT	Pericardial and bilateral pleural effusion, negative for pulmonary embolism (CTPA)	Consolidations in the superior and inferior lobes of the left lung and the inferior lobe of the right lung	NR	Pericardial and left-sided pleural effusion, left side infiltrate 3 × 2 cm	NR	NR
Diagnosis	IgM 20 U/ml (r < 15), IgG 14 (r < 12)–10 days later IgM 11 U/ml (r < 15), IgG 17 (r < 12)–10 days later IgM 11 U/ml (r < 15), IgG 22 (r < 12)	IgM 5.63 (r < 0.9), IgG 1.63 (r < 0.9)–2 weeks later IgM 3.49 (r < 0.9), IgG 2.31 (r < 0.9)	IgM ≥ 1:160 (r < 1:10), IgG ≥ 1:1024 (r < 1:64), IgA ≥ 1:256 (r < 1:16)	Positive IgG and IgA	Positive IgG and IgA, Taq-Man PCR with the pericardial fluid	Cultures and direct immunofluorescence of the pericardial fluid using specific monoclonal amtibodies revealed elementary bodies, IgG 1:64, ΙgM negative
Treatment	Moxifloxacin and ceftriaxone 5 weeks ago for previous CAP, Methylprednisolone, Colchicine	Ceftriaxone 100 mg/kg once daily for 14 days, Clarithromycin 15 mg/kg was added on the third day of ceftriaxone therapy for 10 days	Azithromycin 10 mg/kg once daily for 7 days	Clarithromycin 500 mg 1 × 2 for 6 weeks, Prednisone 60 mg with careful slow tapering	Azithromycin, Cefuroxime, Ibuprofen	Erythromycin 0.5 g 1 × 4
Follow-Up	Discharged after 18 days, follow-up visits every 3–4 weeks, in excellent clinical condition	Discharged after 14 days, follow-up after 2 weeks with new C. pneumoniae IgM and IgG evaluation	Discharged after 9 days (well controlled with anticongestive medication in subsequent visit)	Relapse 4 weeks after stopping treatment; retreated with antibiotic therapy (clarithromycin + metronidazole); now 9 months without symptoms	Discharged after 14 days	Discharged after 14 days; died several months later (first relapse of acute leukemia, intracerebral hemorrhage)

*ANA* antinuclear antibodies, *BNP* brain natriuretic peptide, *bpm* beats per minute, *CAP* community acquired pneumonia, *CRP* C-reactive protein, *CT* computed tomography, *CTPA* computed tomography pulmonary angiography, *ECG* electrocardiography, *ESR* erythrocyte sedimentation rate, *NR* not reported, *RF* rheumatoid factor, *PVCs* premature ventricular contractions, *r* reference, *WBC* white blood cells

**Fig. 1:**
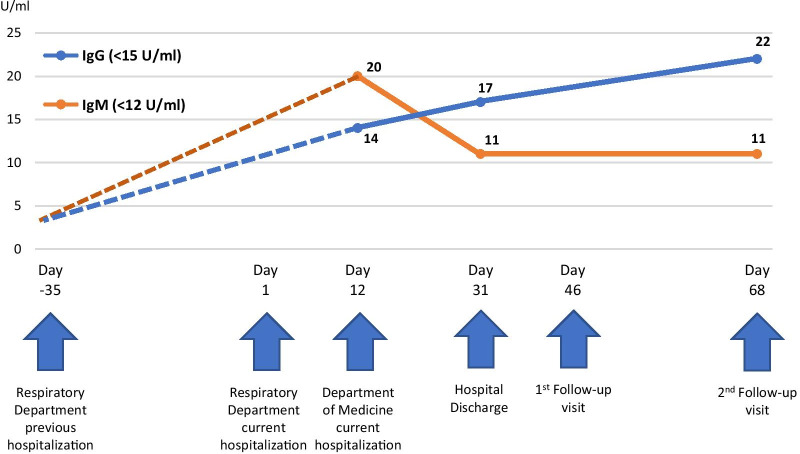
Chlamydia pneumoniae IgM and IgG antibodies kinetics

The patient presented impressive clinical and laboratory response to the combination of colchicine and methylprednisolone and was discharged (D 31) with the diagnosis of *pleuropericarditis under investigation* on colchicine 0.5 mg once daily and methylprednisolone 16 mg twice daily per os, while waiting for further laboratory test results. *C. pneumoniae* IgM and IgG antibodies [enzyme-linked immunosorbent assay (ELISA) VIRION/SERION ELISA kit] were both positive upon admission of the patient to our Department on D 12 (IgM 20 U/ml, IgG 14 U/ml with normal limits < 15 U/ml for IgM and < 12 U/ml for IgG) (Fig. 1). *Mycoplasma pneumoniae* antibody titers were found to be negative, and a possible cross reaction, observed in 30% of patients with *M. pneumoniae* infection, was excluded [16]. In addition, a positive aspergillus antigen test (galactomannan) was found. Clinical and imaging findings were against aspergillosis, the patient was not in an immunodeficient status, and this finding was considered as a false positive finding, probably due to prior treatment with piperacillin/tazobactam [17]. In order to follow the antibody kinetics, new antibody titers were ordered upon patient’s discharge on D 31 (IgM 11 U/ml, IgG 17 U/ml) (Fig. 1). Thus, the combination of the patient’s history and course, the negative extensive work up for other infections and autoimmune diseases, the negative imaging for pulmonary embolism and malignancies, along with the trend of the antibody kinetics rendered a diagnosis of *C. pneumoniae* related pleuropericarditis rather possible.

The patient visited the hospital on an outpatient basis two weeks after discharge (D 46). She was in good clinical condition, with normal laboratory values (white blood cells, CRP) and improved imaging evaluation (mild pleural effusions in CT imaging) (Additional File 1). Tapering of methylprednisolone was decided by reducing 8 mg of methylprednisolone every 7 days. The patient presented to the next scheduled follow-up visit as outpatient one month later (D 68) on colchicine 0.5 mg once daily and methylprednisolone 8 mg once daily. She reported recurrence of mild pleuritic chest pain and low-grade fever. CRP was 2.8 mg/dl (normal limits < 0.7 mg/dl) and imaging (echocardiography) revealed small pericardial effusion. Aggressive tapering of methylprednisolone was considered as the most probable cause of the clinical relapse of the patient. Uptitration of methylprednisolone to 16 mg twice daily was decided. A third sample for *C. pneumoniae* antibodies was acquired (IgM 11 U/ml, IgG 22 U/ml) (Fig. 1). The patient was reassessed on D 105 in good clinical condition and absence of pleural effusion in chest X-ray and closer follow-up was planned for a slower tapering of corticosteroids. Complete rheumatologic evaluation was repeated (clinical and laboratory) without evidence of rheumatological disease.

Concerning the patient’s perspective, she was informed of all the investigations performed and she was compliant with the interventions. She was relieved with the exclusion of severe causes (malignant) and fully aware of the need for regular follow-up. The patient engaged gradually to her daily activities without any concern.

## Discussion and conclusions

The case of a 40-year old woman with pleuropericarditis associated with prior *C. pneumoniae* CAP has been described. Cardiac complications after or during Chlamydia infection (either *C. pneumoniae, psittaci or trachomatis*) have been described through the literature, though cases are rare, and mechanisms understudied. Most data are derived from non-human studies [12], while the association of Chlamydia infection with cardiac complications has been based mainly on cross-sectional studies with single antibody measurements, rather than multiple follow-up antibody titers and kinetics, like in our case [18, 19]. Cardiac involvement seems to be even rarer when it comes to *C. pneumoniae* infection [2–11]. Previous reports associated with *M. pneumoniae* atypical CAP have also been reported and seem to be more common through the literature [20].

**Fig. 2:**
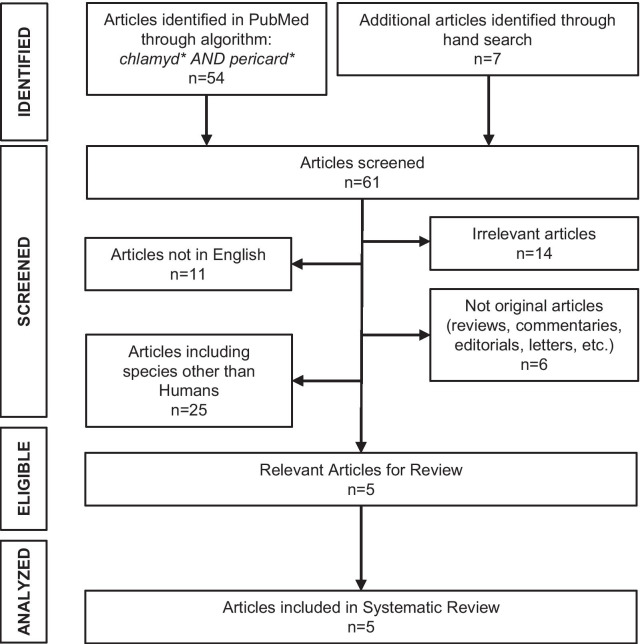
Search algorithm and flowchart of studies included in systematic review

A systematic review of the literature was conducted independently by two investigators (GED, EF) to detect similar cases. Search algorithm and flowchart of included studies are shown in Fig. 2. Similar case reports are rare in the literature. Five studies have been identified [7–11]. Main characteristics of included studies are described in Table 1. The origin of the studies was Europe and Asia, while the prognosis of patients involved was good in most cases. The study by Oztek Celebi et al. [7] was the only one where more than one antibody measurements were ordered (two in total), in order to investigate a similar case involving a 13-year-old boy with pericardial and bilateral pleural effusions. However, antibody titers did not clearly follow the expected kinetics. Our study is unique in this context as more measurements of chlamydial antibodies were ordered (three in total) with typical fluctuations indicating past infection. Regarding treatment choices among the similar studies identified, most patients have been treated mainly with macrolides but there seems to be a lack of evidence regarding the use of colchicine and/or corticosteroids in their treatment (Table 1).

In our case the patient presented to our hospital with pleuropericarditis five weeks after hospitalization for CAP in another hospital, where she was treated with ceftriaxone and moxifloxacin without any report on causative pathogen. The kinetics of the IgM and IgG antibodies against *C. pneumoniae* indicated the latter as the causative microorganism (Fig. 1, Additional File 1). The patient was administered moxifloxacin in her first hospitalization. This is considered as an acceptable first-line empiric treatment for most patients with community-acquired pneumonia since the etiology is usually unknown at the time of diagnosis. The patient responded and was discharged. However, macrolides represent the first-line option for *C. pneumoniae* [21]. Whether an initial course with macrolides could avert the subsequent pericarditis is not known. So far, the documentation and diagnosis of *C. pneumoniae* infection are often suboptimal in terms of standardization and methodological validation. Respiratory tract samples cultures and polymerase chain reaction (PCR) seem to be the most accurate methods, yet high cost and practical problems render them not widely applicable. In the present case, PCR or cultures of the respiratory tract samples would have not probably contributed to the diagnosis due to the delayed occurrence of the serositis after CAP. The delayed emergence of serositis after the infection could indicate indirect (namely autoimmune) mechanisms. Serologic evaluation is the most frequently used method, either by microimmunofluorescence or ELISA. Standard diagnostic criteria for serologic methods apply, such as the fourfold increase of antibody titers within 4–6 weeks after symptoms onset [1, 22]. Unfortunately, we did not have baseline values at symptoms onset and during her first hospitalization. However, the patient’s *C. pneumoniae* antibodies kinetics seem to be in line with our assumption of a recent *C. pneumoniae* CAP. IgM titers presented a peak in our first measurement and then followed a gradual decline, while IgG titers continued to increase within the next 4–6 weeks. (Fig. 1) [1, 22, 23].

Multiple possible mechanisms of cardiac damage have been suggested and could be divided into non-autoimmune and autoimmune mediated pathways [12, 14, 24, 25]. Concerning non-autoimmune pathways, *C. pneumoniae*-infected macrophages seem to present greater adherence to the endothelium and tend to degenerate easier to foam cells in the presence of LDL cholesterol [14, 23]. This suggested mechanism implicates that Chlamydia infection could play a role as a cardiovascular disease (CVD) risk factor for atherosclerosis [14, 24]. Autoimmune mechanisms on the other side have been reported to act mainly via molecular mimicry phenomenon [12, 25]. In this case, chlamydial proteins mimic host self-proteins and may trigger systematic autoimmune reactions [12, 25]. The impressive clinical and biochemistry patient’s response to corticosteroids and the lack of any clinical or laboratory response whenever antibiotics were initiated, is indicative of an autoimmune pathogenetic mechanism.

Our study presents several limitations regarding the diagnostic documentation of patient’s prior chlamydial infection. Appropriate diagnostic evaluation during the patient’s first hospitalization, including PCR/cultures of respiratory tract samples, as well as baseline antibody titers, was unavailable; however, such a diagnostic work-up is not routinely recommended for uncomplicated CAP. It should be mentioned that the patient presented a rather late serositis a few weeks after her discharge and remission of her initial symptoms. Indeed, similar limitations have been encountered in all relevant published studies (Table 1) [7–11]. These ‘inevitable’ limitations are somewhat expected in the setting of everyday clinical practice and real-world medicine. Assessment of antibody kinetics in this study might at least partly counteract some of these limitations. In fact, the etiology of polyserositis was determined on the basis of dynamic changes in antibody levels, the clinical course and the response of the patient to steroid and colchicine therapy, and the exclusion of other etiologies. It should be taken into consideration that the aim of this case presentation was to describe a probable rather than definite causative relation of chlamydial infection and pericarditis, and most importantly to raise awareness among doctors regarding the early consideration of this entity in differential diagnosis of similar cases.

In conclusion, *C. pneumoniae*-associated CVD or autoimmune implications may be more frequent and important than generally thought. Clinicians should be aware of atypical causes in the absence of profound and obvious explanations. Nonsteroidal anti-inflammatory drugs and colchicine constitute the mainstay of therapy in cases of pericarditis. Corticosteroids should be considered in cases of contraindications and/or failure of the above-mentioned regimens or in cases of autoimmune-related serositis.

## Supplementary Information

**Additional file 1**: Graphical presentation of the case report including imaging findings, laboratory findings, and therapeutic strategy.

## Data Availability

The original contributions presented in the study are included in the article/supplementary material. Further inquiries can be directed to the corresponding author.

## Abbreviations

CAP: Community acquired pneumonia
CVD: Cardiovascular disease
CRP: C-reactive protein
CT: Computed tomography
D: Day
ELISA: Enzyme-linked immunosorbent assay

## References

[1] Sharma L, Losier A, Tolbert T, Dela Cruz CS, Marion CR (2017). Atypical pneumonia: updates on legionella, chlamydophila, and mycoplasma pneumonia. Clin Chest Med.

[2] Gran JT, Hjetland R, Andreassen AH (1993). Pneumonia, myocarditis and reactive arthritis due to Chlamydia pneumoniae. Scand J Rheumatol.

[3] Fairley CK, Ryan M, Wall PG, Weinberg J (1996). The organisms reported to cause infective myocarditis and pericarditis in England and Wales. J Infect.

[4] Gnarpe H, Gnarpe J, Gästrin B, Hallander H (1997). Chlamydia pneumoniae and myocarditis. Scand J Infect Dis Suppl.

[5] Odeh M, Oliven A (1992). Chlamydial infections of the heart. Eur J Clin Microbiol Infect Dis.

[6] Tong CY, Potter F, Worthington E, Mullins P (1995). Chlamydia pneumoniae myocarditis. Lancet.

[7] Oztek Celebi FZ, Fettah A, Yesil S (2020). Acute haemorrhagic pericarditis: an unusual presentation of Chlamydophila pneumoniae pneumonia infection. Paediatr Int Child Health.

[8] Suesaowalak M, Cheung MM, Tucker D, Chang AC, Chu J, Arrieta A (2009). Chlamydophila pneumoniae myopericarditis in a child. Pediatr Cardiol.

[9] Rýzlová M, Gregor P (2008). Acute pericarditis as an organic manifestation of the acute infection Chlamydia pneumoniae. Vnitr Lek.

[10] Tenenbaum T, Heusch A, Henrich B, MacKenzie CR, Schmidt KG, Schroten H (2005). Acute hemorrhagic pericarditis in a child with pneumonia due to Chlamydophila pneumoniae. J Clin Microbiol.

[11] Zver S, Kozelj M, Cernelc P (1997). Chlamydia pneumoniae pneumonia with acute hemorrhagic pericarditis in patient with acute leukemia. Haematologica.

[12] Bachmaier K, Penninger JM (2005). Chlamydia and antigenic mimicry. Curr Top Microbiol Immunol.

[13] Joshi R, Khandelwal B, Joshi D, Gupta OP (2013). Chlamydophila pneumoniae infection and cardiovascular disease. N Am J Med Sci.

[14] Campbell LA, Kuo CC, Grayston JT (1998). Chlamydia pneumoniae and cardiovascular disease. Emerg Infect Dis.

[15] Adler Y, Charron P, Imazio M, et al; ESC Scientific Document Group. 2015 ESC Guidelines for the diagnosis and management of pericardial diseases: The Task Force for the Diagnosis and Management of Pericardial Diseases of the European Society of Cardiology (ESC) Endorsed by: The European Association for Cardio-Thoracic Surgery (EACTS). Eur Heart J. 2015;36(42):2921–64. 10.1093/eurheartj/ehv318. 10.1093/eurheartj/ehv318 PMC7539677 26320112

[16] Miyashita N, Akaike H, Teranishi H, et al; Atypical Pathogen Study Group. Chlamydophila pneumoniae serology: cross-reaction with Mycoplasma pneumoniae infection. J Infect Chemother. 2013;19(2):256–60. 10.1007/s10156-012-0494-4. 10.1007/s10156-012-0494-4 23065148

[17] Boonsarngsuk V, Niyompattama A, Teosirimongkol C, Sriwanichrak K (2010). False-positive serum and bronchoalveolar lavage Aspergillus galactomannan assays caused by different antibiotics. Scand J Infect Dis.

[18] Danesh J, Collins R, Peto R (1997). Chronic infections and coronary heart disease: is there a link?. Lancet.

[19] Mahdi OS, Horne BD, Mullen K, Muhlestein JB, Byrne GI (2002). Serum immunoglobulin G antibodies to chlamydial heat shock protein 60 but not to human and bacterial homologs are associated with coronary artery disease. Circulation.

[20] Vijay A, Stendahl JC, Rosenfeld LE (2019). Mycoplasma pneumoniae pericarditis. Am J Cardiol.

[21] Centers for Disease Control and Prevention. Chlamydia pneumoniae Infection/Treatment. https://www.cdc.gov/pneumonia/atypical/cpneumoniae/hcp/treatment.html (2019). Accessed 25 June 2021.

[22] Hvidsten D, Halvorsen DS, Berdal BP, Gutteberg TJ (2009). Chlamydophila pneumoniae diagnostics: importance of methodology in relation to timing of sampling. Clin Microbiol Infect.

[23] Miyashita N, Kawai Y, Tanaka T (2015). Antibody responses of Chlamydophila pneumoniae pneumonia: why is the diagnosis of C. pneumoniae pneumonia difficult?. J Infect Chemother.

[24] Stassen FR, Vainas T, Bruggeman CA (2008). Infection and atherosclerosis. An alternative view on an outdated hypothesis. Pharmacol Rep.

[25] Díaz F, Collazos J (1997). Myopericarditis due to Chlamydia psittaci. The role of autoimmunity. Scand J Infect Dis.

