# Supplementary material for: Chlamydia pneumoniae-associated pleuropericarditis: a case report and systematic review of the literature
Source: BMC Pulm Med. 2021 Nov 22;21:380. doi: 10.1186/s12890-021-01743-9 (PMC8607726; doi:10.1186/s12890-021-01743-9)
Supplement: Supplementary file 1 — Additional file 1: Graphical presentation of the case report including imaging findings, laboratory findings, and therapeutic strategy. [file 12890_2021_1743_MOESM1_ESM.pptx]

## Slide 1
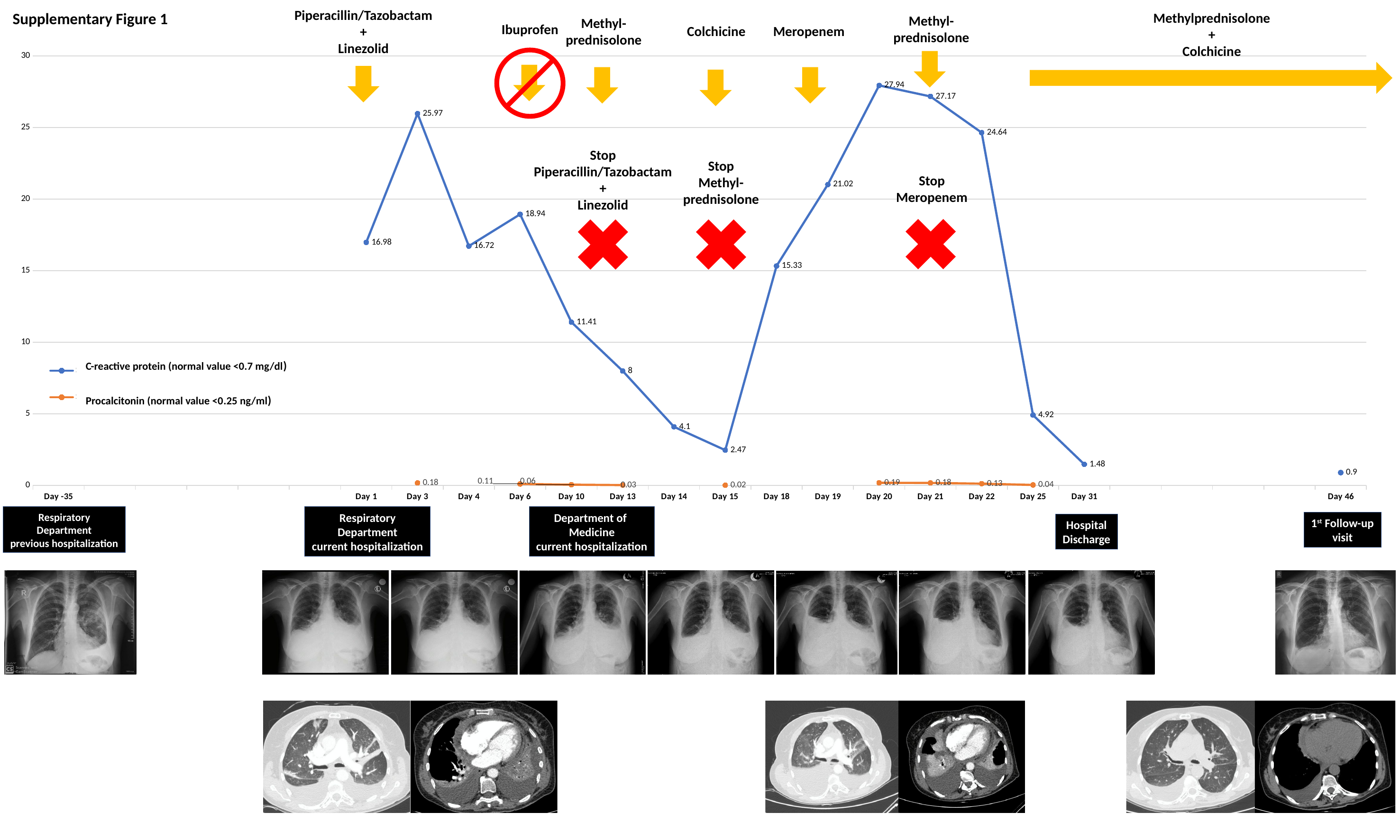

Piperacillin/Tazobactam
+
Linezolid
Supplementary Figure 1
Methylprednisolone
 +
Colchicine
Methyl-
prednisolone
Methyl-
prednisolone
Ibuprofen
Colchicine
Meropenem
### Chart
| Category | | |
|---|---|---|
| Day -35 | None | None |
| | None | None |
| | None | None |
| | None | None |
| | None | None |
| | None | None |
| Day 1 | 16.98 | None |
| Day 3 | 25.97 | 0.18 |
| Day 4 | 16.72 | None |
| Day 6 | 18.94 | 0.11 |
| Day 10 | 11.41 | 0.06 |
| Day 13 | 8.0 | 0.03 |
| Day 14 | 4.1 | None |
| Day 15 | 2.47 | 0.02 |
| Day 18 | 15.33 | None |
| Day 19 | 21.02 | None |
| Day 20 | 27.94 | 0.19 |
| Day 21 | 27.17 | 0.18 |
| Day 22 | 24.64 | 0.13 |
| Day 25 | 4.92 | 0.04 |
| Day 31 | 1.48 | None |
| | None | None |
| | None | None |
| | None | None |
| | None | None |
| Day 46 | 0.9 | None |Stop
Piperacillin/Tazobactam
+
Linezolid
Stop
Methyl-
prednisolone
Stop
Meropenem
C-reactive protein (normal value <0.7 mg/dl)
Procalcitonin (normal value <0.25 ng/ml)
Respiratory
Department
previous hospitalization
Respiratory
Department
current hospitalization
Department of
Medicine
current hospitalization
1st Follow-up
visit
Hospital
Discharge
